# Association between epidermal growth factor receptor mutations and the expression of excision repair cross-complementing protein 1 and ribonucleotide reductase subunit M1 mRNA in patients with non-small cell lung cancer

**DOI:** 10.3892/etm.2015.2196

**Published:** 2015-01-21

**Authors:** CHUN-WEI XU, GANG WANG, WU-LONG WANG, WEN-BIN GAO, CHUAN-JUN HAN, JING-SHAN GAO, YANG LI, LIN WANG, LI-YING ZHANG, YU-PING ZHANG, YU-WANG TIAN, JIN-NV FANG

**Affiliations:** 1 Department of Pathology, The General Military Hospital of Beijing PLA, Beijing 100700, P.R. China; 2 Department of Oncology, Affiliated Zhongshan Hospital of Dalian University, Dalian, Liaoning 116001, P.R. China; 3 Graduate School of Dalian University, Dalian, Liaoning 116622, P.R. China; 4 The Second Affiliated Hospital of Baotou Medical College, Baotou, Inner Mongolia 014030, P.R. China; 5 Department of Clinical Medicine, Dalian Medical University, Dalian, Liaoning 116044, P.R. China; 6 Department of Oncology, The General Military Hospital of Beijing PLA, Beijing 100700, P.R. China; 7 Department of Pathology, Shanxi Da Hospital, Shanxi Academy of Medical Sciences, Taiyuan, Shanxi 030001, P.R. China; 8 Department of Pathology, The People’s Hospital of Weifang, Weifang, Shandong 261041, P.R. China; 9 Department of Medicine, Yanbian University, Yanji, Jilin 133002, P.R. China

**Keywords:** non-small cell lung cancer, epidermal growth factor receptor, excision repair cross-complementing protein 1, ribonucleotide reductase subunit M1, molecular detection, individualized treatment

## Abstract

The present study aimed to investigate the association between epidermal growth factor receptor (*EGFR*) gene mutations and excision repair cross-complementing protein 1 (*ERCC1*) and ribonucleotide reductase subunit M1 (*RRM1*) mRNA expression in non-small cell lung cancer (NSCLC) tissue. The quantitative polymerase chain reaction was used to detect *EGFR* mutations, and *ERCC1* and *RRM1* mRNA expression in 257 cases of NSCLC. In the NSCLC samples the *EGFR* mutation rate was 49.03% (126/257). The rate was higher in females and non-smoking patients (P<0.05). High expression of *ERCC1* mRNA was observed in 47.47% of the samples (122/257), while a high *RRM1* mRNA expression was observed in 61.87% of the samples (159/257). In comparison with patients with NSCLC without *EGFR* mutations, patients with *EGFR* mutations had significantly lower levels of *ERCC1* mRNA expression (P<0.05); however, *EGFR* mutations and expression levels of *RRM1* mRNA were not correlated in NSCLC tissues (P>0.05). In addition, *ERCC1* mRNA expression was not correlated with the expression levels of *RRM1* mRNA (P>0.05). In conclusion, patients with NSCLC with *EGFR* mutations tend to have a low expression of *ERCC1* mRNA and may potentially benefit from platinum-based chemotherapy.

## Introduction

Globally, lung cancer has the highest rates of morbidity and mortality of all malignancies (1). Non-small cell lung cancer (NSCLC) accounts for 80–85% of lung cancer cases worldwide (2).

Human epidermal growth factor receptor (EGFR) belongs to the type I receptor family. This family has four cognate family members, including EGFR (HER1), HER2, HER3 and HER4, which mediate the following signal transduction pathways: Ras-Raf-mitogen-activated protein kinase kinase-extracellular signal-regulated kinase-mitogen-activated protein kinase, phospholipase C-γ, phosphatidylinositol-3-kinase/phosphoinositide-dependent kinase 1 and Janus kinase/signal transducers and activators of transcription (3). These receptors regulate cell proliferation, differentiation and apoptosis (4). *EGFR* mutations in patients with NSCLC occur in the intracellular tyrosine kinase (TK) region above the first four exons (18 to 21). A total of 30 different types of mutations have been identified in the TK region (5), the most common occurring in exons 19 and 21, which account for ~85% of all the mutations (6).

Excision repair cross-complementing gene 1 (*ERCC1*) is the key gene in two DNA repair pathways: nucleotide excision repair (NER) and chain crosslink repair (7). Overexpression of *ERCC1* can rapidly repair damaged DNA arrest at the G_2_/M phase and cause cells to be resistant to platinum (8). Ribonucleotide reductase subunit M1 (*RRM1*) is involved in DNA synthesis and repair (9). Results from the Iressa^®^ Pan-Asia Study (10) clinical trial indicated that, in Asian populations, patients with *EGFR* mutations were more responsive to chemotherapy than patients with wild-type *EGFR.* Patients with lung cancer with low expression of *ERCC1* and *RRM1* are more responsive to gemcitabine- and platinum-based chemotherapy, respectively (11,12). Patients with NSCLC are generally treated with chemotherapy drugs, including cisplatin and gemcitabine. In the present study, 257 patients with stages I-IV NSCLC from multiple hospitals were analyzed for the presence of *EGFR* mutations and expression levels of *ERCC1* and *RRM1* mRNA. The data were statistically analyzed to determine significant correlations between *EGFR* mutations and expression of these two chemotherapy resistance genes in patients with NSCLC. These data may prove useful in further identifying more effective individualized treatment plans for patients with *EGFR* mutations, particularly for patients with small-molecule EGFR-tyrosine kinase inhibitor (EGFR-TKI) primary or secondary resistance.

## Materials and methods

### Specimens

For the detection of *EGFR* mutations, as well as *ERCC1* and *RRM1* mRNA expression levels, paraffin tissue specimens were collected from 257 patients from the General Military Hospital of Beijing PLA (Beijing, China; 103 cases), the Affiliated Zhongshan Hospital of Dalian University (Dalian, China; 58 cases) and the People’s Hospital of Weifang (Weifang, China; 96 cases). The patients had undergone surgery between 2004 and 2013; the pathological diagnosis was adenocarcinoma, and patients had not received preoperative chemotherapy, radiotherapy or biological immunotherapy. All protocols in the present study were approved by the Human Clinical and Research Ethics Committees of the General Military Hospital of Beijing PLA, the Affiliated Zhongshan Hospital of Dalian University (Dalian, China) and the People’s Hospital of Weifang (Weifang, China). Written informed consent was obtained from all of the patients.

### Reagents and instruments

The DNA and RNA extraction kits were purchased from Qiagen (Hilden, Germany). The human *EGFR* mutation qualitative detection kit and the tumor-related gene expression relative quantification detection kit (*ERCC1* and *RRM1*) were obtained from Amoy Diagnostics Co., Ltd. (Xiamen, China). The B-500 instrument to measure nucleic acid protein concentrations was purchased from Shanghai Chong Meng Biotechnology Co. Ltd. (Shanghai, China) and the ABI 7500 quantitative polymerase chain reaction (qPCR) instrument was purchased from Applied Biosystems^®^ (Life Technologies, Foster City, CA, USA).

### qPCR to detect EGFR mutations in NSCLC tissues

**Figure 1:**
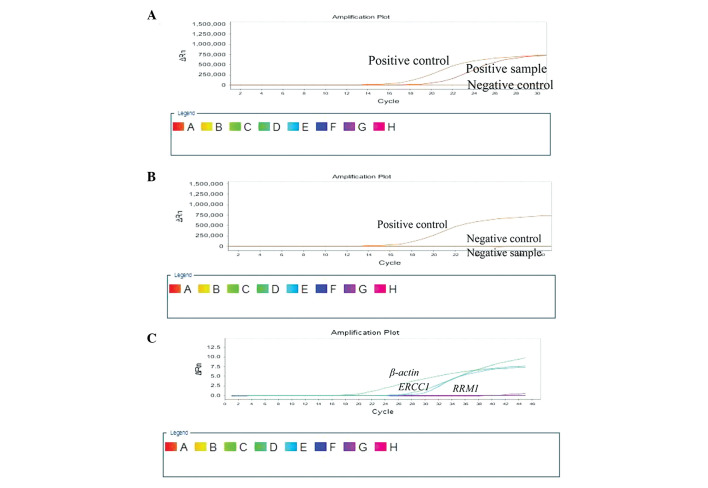
Mutations of the *EGFR* gene and the expression of *ERCC1* and *RRM1* mRNA. (A) Positive control, positive sample and negative control of the *EGFR* gene. (B) Positive control, negative sample and negative control of the *EGFR* gene. (C) Expression of *ERCC1* and *RRM1* mRNA. *EGFR*, epidermal growth factor receptor; *ERCC1*, excision repair cross-complementing protein 1; *RRM1*, ribonucleotide reductase subunit M1.

Between four and eight 4-μm-thick paraffin tissue sections were obtained and dewaxed. The genomic RNA extraction kit was used to extract RNA from the tissue samples according to the manufacturer’s instructions. A spectrophotometer was used to determine the purity and concentration of the extracted RNA, which was used as a template to synthesize the corresponding DNA. The human *EGFR* mutation qualitative detection kit, which contains 29 different fusion mutant primers and probes to amplify *EGFR* exons 18, 19, 20 and 21, was used (ADx-EG09; Amoy Diagnostics Co., Ltd.); the DNA was amplified in an ABI 7500 qPCR instrument (Fig. 1A and B). The qPCR cycling conditions were set as follows: 95°C for 5 min, follwed by 45 cycles of 95°C for 30 sec, 60°C for 30 sec and 72°C for 45 sec.

### qPCR to detect the expression of ERCC1 and RRM1 mRNA in NSCLC tissue

RNA was extracted from 4-μm-thick paraffin tissue sections in accordance with the aforementioned methods. The tumor-associated gene expression detection kit for *ERCC1* (ADx-ER01) and *RRM1* (ADx-RR01) (Amoy Diagnostics Co., Ltd.) was used to determine the mRNA expression level of these genes using the absolute quantitative method; β-actin was used as the reference gene. *ERCC1* had a standard mean of 4.29×10^−3^, and *RRM1* had a standard mean of 11.37×10^−3^ (Fig. 1C).

### Statistical analysis

The data were analyzed using the statistical software SPSS (version 19.0; IBM SPSS, Armonk, NY, USA) using the χ^2^ and Fisher’s exact test with a test level α=0.05. The P-value was set to bilateral distribution and P<0.05 was considered to indicate a statistically significant difference.

## Results

### Association between EGFR mutations, expression of ERCC1 and RRM1 mRNA, and patient clinical characteristics

Of the 257 cases of NSCLC, *EGFR* mutations were present in 126 cases (49.03%). The *EGFR* mutation rate was higher in non-smoking patients (92/158, 58.23%; P<0.001); however, the *EGFR* mutation rate was not associated with the age, tumor size, lymph-node metastasis or clinical stage of the patient. High expression of *ERCC1* mRNA was observed in 122/257 cases (47.47%). *ERCC1* mRNA expression levels were not associated with the gender, age, smoking status, tumor size, lymph-node metastasis or clinical stage of the patient. High expression of *RRM1* mRNA was observed in 159/257 cases (61.87%). *RRM1* mRNA expression levels were not associated with the gender, age, smoking status, tumor size, lymph-node metastasis or clinical stage of the patient (Table I).

### Association between EGFR mutations and expression levels of ERCC1 mRNA

Of the 126 patients with NSCLC with an *EGFR* mutation, 79 (62.70%) showed low expression of *ERCC1*. Of the 131 patients with NSCLC with the wild-type *EGFR* gene, 56 (42.75%) had low expression of *ERCC1* mRNA. These data indicate that patients with NSCLC with an *EGFR* mutation had significantly lower expression of *ERCC1* mRNA (P<0.05) (Table II).

### Association between EGFR mutations and the expression level of RRM1 mRNA

Of the 126 patients with NSCLC with an *EGFR* gene mutation, 51 (40.48%) had low expression of *RRM1* mRNA, while 47 out of the 131 patients (35.88%) with the wild-type *EGFR* gene had low expression of RRM1 mRNA. These data indicate that there was no correlation between *EGFR* and *RRM1* mutations in patients with NSCLC (P>0.05) (Table III).

### Association between the expression levels of ERCC1 and RRM1 mRNA

Of the 122 patients with NSCLC with high expression of *ERCC1* mRNA, 72 cases (59.02%) had high expression of *RRM1* mRNA. Of the 135 patients with NSCLC with low expression of *ERCC1* mRNA, 48 cases (35.56%) had low expression of *RRM1* mRNA. A total of 120 cases had low or high expression of both *ERCC1* and *RRM1* mRNA, and 137 cases had contrasting expression levels of *ERCC1* and *RRM1* mRNA. These data indicate that the expression of *ERCC1* and *RRM1* mRNA was not significantly correlated (χ^2^=0.800, P=0.371) in NSCLC tissue (Table IV).

## Discussion

*EGFR* mutations and the responsiveness of NSCLC to the molecular targeted drugs gefitinib (trade name, Iressa) and erlotinib (trade name, Tarceva^®^) have a close association (13,14). Small-molecule TKIs have been shown to have a high efficiency in patients with an exon 19 deletion in the *EGFR* gene (15); however, patients with NSCLC with *EGFR* mutations in exon 20 are resistant to drug treatment with TKIs (16). Other studies have reported that the NER complexes prognosis is good but not suitable for receiving platinum-based chemotherapy (17,18).

The results of the present study showed that the *EGFR* mutation rate was 48.03% (126/257) in patients with stages I-IV NSCLC, which is consistent with previously reported data (14,19). A higher percentage of patients with NSCLC (47.47%; 122/257) showed high mRNA expression levels of *ERCC1* compared with data from a previous study (20), while the percentage of patients with NSCLC with a high *RRM1* mRNA expression level (61.87%; 159/257) was consistent with data presented in a previous study (21). The expression levels of *ERCC1* and *RRM1* mRNA were not associated with the gender, age, smoking status, tumor size, lymph node metastasis, pathological staging or other clinical characteristics of the patient.

The current study found that the mutational status of *EGFR* was associated with *ERCC1* mRNA expression levels in patients with NSCLC; patients with *EGFR* mutations had a significantly lower expression of *ERCC1* mRNA (P<0.05). *EGFR* mutations did not, however, significantly correlate with *RRM1* expression levels (P>0.05) in patients with NSCLC. A previous study has shown that *EGFR* mutations in patients with NSCLC were correlated with expression levels of *ERCC1* (P<0.001). Furthermore, *EGFR* mutations in the adenocarcinoma subgroup were correlated with *ERCC1* expression levels (P=0.001) (22). It has also been shown that NER enzymes in cells can lead to cell damage, resulting in genomic instability and an increased mutation rate (23). Cancer cells with low expression of *ERCC1* have a decreased ability to repair DNA damage, an increase in the number of *EGFR* mutations and increased sensitivity to platinum-based chemotherapy, which may be why patients with NSCLC with *EGFR* mutations have a higher response rate to chemotherapy.

The present study also found that, in NSCLC tissues, mRNA expression levels of *ERCC1* and *RRM1* were not correlated (P>0.05), which is inconsistent with data presented by Reynolds *et al* (24). The association between the expression of *ERCC1* and *RRM1* mRNA remains controversial and warrants further research. At present, numerous studies have confirmed that therapy can be selected based on patient expression levels of *ERCC1* and *RRM1,* and this can be extended to patients with NSCLC (25,26).

In conclusion, the current study has demonstrated that patients with *EGFR* mutations tend to have lower expression levels of *ERCC1* mRNA. We hypothesize that patients with *EGFR* mutations may be more responsive to cisplatin-based chemotherapy, although the molecular mechanisms require further study. No difference in the expression levels of *RRM1* mRNA was observed between patients with NSCLC with *EGFR* mutations and those without mutations. In the present study, we have determined the first-line chemotherapy for tumors involving platinum and gemcitabine drug resistance genes and *EGFR* mutations but not the microtubule drug resistance gene *TUBB3* or the thymidylate synthase resistance gene *TYMS*. Future studies, therefore, are likely to focus on *EGFR*, *TUBB3* and *TYMS* mutations to better identify effective individualized treatment plans, particularly individualized treatment plans for EGFR-TKI (e.g. imatinib) primary or secondary resistance observed in certain patients.

## Figures and Tables

**Table I:** Association between *EGFR* mutations, *ERCC1* and *RRM1* mRNA expression, and patient clinical characteristics.

	*EGFR*			*ERCC1*			*RRM1*		
						
Clinical features	Mutant (n)	Wild-type (n)	P-value	High (n)	Low (n)	P-value	High (n)	Low (n)	P-value
Gender			<0.001			0.898			0.088
Male	52	86		65	73		92	46	
Female	74	45		57	62		67	52	
Age, years			0.287			0.943			0.714
≥59	68	62		62	68		79	51	
<59	58	69		60	67		80	47	
Smoking history			<0.001			0.199			0.129
Yes	34	65		52	47		67	32	
No	92	66		70	88		92	66	
Tumor diameter, cm			0.282			0.687			0.292
≥5	56	67		60	63		72	51	
<5	70	64		62	72		87	47	
Lymph node metastasis			0.903			0.470			0.188
Yes	51	54		47	58		70	35	
No	75	77		75	77		89	63	
Clinical stage			0.203			0.339			0.577
I	50	42		40	52		59	33	
II+III+IV	76	89		82	83		100	65	

*EGFR*, epidermal growth factor receptor; *ERCC1*, excision repair cross-complementing protein 1; *RRM1*, ribonucleotide reductase subunit M1.

**Table II:** Association between *EGFR* mutations and the expression level of *ERCC1* mRNA.

	*ERCC1* mRNA expression		
		
*EGFR*	High (n)	Low (n)	Total (n)
Mutant	47	79	126
Wild-type	75	56	131
Total (n)	122	135	257

*EGFR*, epidermal growth factor receptor; *ERCC1*, excision repair cross-complementing protein 1.

**Table III:** Association between *EGFR* mutations and the expression level of *RRM1* mRNA.

	*RRM1* mRNA expression		
		
*EGFR*	High (n)	Low (n)	Total (n)
Mutant	75	51	126
Wild-type	84	47	131
Total (n)	159	98	257

*EGFR*, epidermal growth factor receptor; *RRM1*, ribonucleotide reductase subunit M1.

**Table IV:** Association between the expression levels of *ERCC1* and *RRM1* mRNA.

	*RRM1* mRNA expression		
		
*ERCC1* mRNA expression	High (n)	Low (n)	Total (n)
High	72	50	122
Low	87	48	135
Total (n)	159	98	257

*ERCC1*, excision repair cross-complementing protein 1; *RRM1*, ribonucleotide reductase subunit M1.
